# A Qualitative Study Exploring How the Perspectives and Experiences of Cisgender Black Women Inform Their Readiness to Consider Pre-Exposure Prophylaxis for HIV Prevention

**DOI:** 10.3390/ijerph22040558

**Published:** 2025-04-03

**Authors:** Mandy J. Hill, Amber I. Sophus, Aaliyah Gray, Jaylen I. Wright

**Affiliations:** 1 Department of Population Health and Health Disparities, School of Public and Population Health, University of Texas Medical Branch at Galveston (UTMB), Galveston, TX 77555, USA; majhill@utmb.edu; 2 Department of Health Promotion and Disease Prevention, Robert Stempel College of Public Health & Social Work, Florida International University, Miami, FL 33199, USA; 3 Center for Women’s and Gender Studies, College of Arts, Science and Education, Florida International University, Miami, FL 33199, USA; aagray@fiu.edu; 4 American Heart Association, Dallas, TX 75321, USA; jaylen.wright@heart.org

**Keywords:** pre-exposure prophylaxis, PrEP, HIV, readiness, perceived risk, facilitators, barriers, healthcare system, women’s health

## Abstract

Attention to increasing pre-exposure prophylaxis (PrEP) use among cisgender Black women (CBW) in the southern United States (U.S.) is necessary to achieve national 2030 Ending the HIV Epidemic (EHE) goals. Qualitative exploration of CBW’s readiness to use PrEP is necessary to discern whether practical solutions to addressing PrEP uptake within this HIV-vulnerable population are feasible. Focus group discussions (n = 5) and key informant interviews (n = 3) in two EHE jurisdictions in Houston and Austin, Texas were used to explore how perspectives and lived experiences may serve as facilitators and/or barriers to PrEP readiness among 20 CBW. Codes highlighted facilitators and barriers to PrEP readiness. Facilitators involved positive experiences with the healthcare system, high perceived HIV vulnerability, and prioritizing PrEP as self-care. Barriers encompassed concerns with sexual relationship dynamics, mental health implications, and access to humane treatment within the healthcare system. High perceived vulnerability of HIV acquisition is related to an awareness that CBW may not know the entirety of their partner’s sexual activities. Findings indicate precursors of PrEP readiness and challenge the notion that CBW have low perceived vulnerability of acquiring HIV.

## 1. Introduction

Human Immunodeficiency Virus (HIV) presents a persistent and profound public health challenge for cisgender Black women (CBW), who experience a disproportionately higher rate of new HIV cases compared to women of other racial and ethnic backgrounds [1,2,3]. Nationally, CBW account for 50% of HIV diagnoses and are 14.6 times more likely to acquire HIV compared to their White counterparts [1,2,3]. Regionally, this disparity is more pronounced in Southern states like Texas, where Black women comprise 56% of women living with HIV despite representing only 13% of the state’s female population [4,5]. Houston and Austin, two of Texas’ largest cities with significant Black female populations [6], face high cases of HIV and acquired immune deficiency syndrome (AIDS) diagnoses and people living with HIV [7]. Houston’s Harris County, in particular, has high HIV incidence rates. In 2021, Harris County had one of the highest HIV incidence rates (rate = 24.5 per 100,000, cases = 1158) in the state [8], with 28,659 individuals living with HIV (rate = 606.2 per 100,000) [7]. Black women in this county represent the second highest racial group diagnosed with HIV among all women [7,8]. This trend is fueled by a complex interplay of social determinants, structural inequities, and systemic barriers, all contributing to the elevated vulnerability of HIV transmission among CBW [9]. Some of the reasons that contribute to the imbalance in HIV vulnerability among Black women, compared to other women, result from behaviors that include condomless heterosexual sex with non-monogamous partners, coerced or involuntary sex, and transactional sex (e.g., sex for money, drugs, shelter) [10,11]. Gender-based inequities, relationship power and control differentials, and actual or threatened physical or sexual violence are associated with reasons to engage in these behaviors and also serve as reasons to avoid use of effective HIV prevention strategies [12,13,14,15,16,17,18]. Given these findings, there is an urgent need for targeted interventions, particularly with enhancing pre-exposure prophylaxis (PrEP) uptake within this community of women.

PrEP is an FDA-approved antiretroviral medication, available as a daily oral pill [19,20] or a bi-monthly intramuscular injection [19,20,21], that reduces vulnerability to HIV acquisition among HIV-negative individuals [22,23,24,25,26,27,28,29,30]. Research indicates that daily oral PrEP is 99% effective in preventing HIV transmission through sexual activity, yet only an estimated 2% of PrEP-eligible Black women utilize this prevention method [31,32,33]. PrEP prescriptions are lowest in Southern states like Texas and states with higher proportions of Black residents [8,34]. Given that PrEP has been FDA-approved since 2012 and has been properly tested on women globally [22,23,29,30], PrEP marketing campaigns have often failed to adequately include or represent CBW [35,36]. Promotional efforts primarily target sexual minority men and transgender individuals [37,38,39]. This lack of representation may contribute to CBW’s lower PrEP awareness and higher rates of misinformation about PrEP [40,41,42,43,44,45], inadvertently leading CBW to perceive PrEP as an unsuitable HIV prevention method for them [43,46,47,48]. Alternatively, once informed of the usefulness and effectiveness of PrEP, most CBW find the option appealing [43,49].

Additional barriers include low perceived HIV vulnerability, misconceptions related to PrEP, costs associated with accessing and using PrEP, concerns about side effects and drug safety, PrEP-related stigma, structural barriers (i.e., lack of access to preventive sexual health services, healthcare, education [50,51]), and medical mistrust [52,53,54,55,56,57,58] or distrust in the medical system [56]. Medical mistrust is a general sense of suspicion that is likely linked to a group identity and may be a personal or vicarious experience of medical racism from the healthcare system [49,52,59,60]. Medical distrust is a direct and overt expectation that the medical system is likely to act in a way that will do personal harm to an individual and others in that individual’s identity group [56,61]. Both medical mistrust and distrust in the medical system remain a significant concern for CBW, and these constructs continue to impede this group’s readiness and willingness to fully engage in HIV preventive care and/or treatment [43,55,56,62,63]. For instance, studies have shown that heightened medical mistrust can decrease the likelihood of patient–provider communication about vulnerable sexual behaviors and/or discussions about sexual health resources, like PrEP, and as such, limit PrEP awareness, access, and uptake [49,51,56,64,65]. Addressing medical mistrust and distrust may enhance patient–provider communication about PrEP and thus help to increase uptake among CBW [49]. However, common obstacles to effective patient–provider communication about PrEP include providers’ lack of knowledge about PrEP, perceived stigma from healthcare providers regarding substance misuse and/or sexual promiscuity, and insufficient continuity of care [66].

Intervention efforts have been made to address these barriers [36,57,67,68,69,70]. For instance, social media marketing strategies [36,71] and a salon-based intervention (which used a narrative-based education video series) [72,73] have been used to increase PrEP awareness and knowledge among CBW. A brief culturally tailored intervention, MI-PrEP, incorporated motivational interviewing strategies to improve PrEP knowledge, motivation to use, and actual use of PrEP [74]. A combination of evidence-based strategies, such as informational, motivational, and reminder messaging; peer support for HIV prevention; and strengths-based, goal-focused, and problem-solving telehealth coaching delivered by peer paraprofessionals have been used to address PrEP barriers and increase PrEP use and sustained use over time [68]. Involving the community in developing, designing, and implementing interventions for effectiveness has also been found to improve the distribution of PrEP information and PrEP care among CBW [70].

While the identification of barriers and facilitators is useful for intervention development, CBW may be at different stages in their lives, with varying levels of awareness, perception of personal HIV vulnerability, and access to healthcare resources. These factors (and others) can significantly impact CBW’s PrEP readiness—an individual’s willingness, ability, and preparedness to fully engage in the PrEP care continuum that includes (1) identifying individuals who are most vulnerable to contracting HIV, (2) increasing awareness of HIV vulnerabilities among those who are most vulnerable, (3) enhancing PrEP awareness, (4) facilitating PrEP access, (5) linking to PrEP care, (6) prescribing PrEP, (7) initiating PrEP, (8) adhering to PrEP, and (9) retaining individuals in PrEP care [75]. For instance, a woman experiencing stable healthcare access and supportive social networks might be more prepared to start PrEP compared to someone with medical mistrust or facing financial constraints. Thus, understanding the factors that facilitate or hinder readiness to adopt PrEP for HIV prevention is a more realistic and vital step toward reducing the reasons for HIV vulnerability within populations of CBW. To conceptualize PrEP readiness related to the steps required for PrEP initiation, the PrEP care continuum [75,76] provides a useful framework when integrated with the Transtheoretical Model of Change (TTM) [77].

The PrEP care continuum outlines the sequential steps individuals go through when accessing and utilizing PrEP [75,76,78,79]. When analyzed through the lens of the TTM [77], the PrEP care continuum provides a structured framework for understanding an individual’s readiness to initiate PrEP. The TTM frames behavior change through decisions made over time and suggests that behavior change occurs in 5 stages: pre-contemplation (no intention to change), contemplation (considering change), preparation (getting ready to change), action (actively making the change), and maintenance (sustaining the change) [77]. Within the context of the PrEP care continuum, PrEP-eligible individuals in the PrEP pre-contemplation stage (Stage 1) do not consider themselves to be vulnerable to HIV and/or are not willing to initiate PrEP [76]. In the PrEP contemplation stage (Stage 2), individuals recognize their potential need for PrEP but have not yet made plans or taken steps to obtain it [76]. Those in the PrEParation stage (Stage 3) have identified how to access PrEP and are making plans to initiate it but have not yet received a PrEP prescription [76]. Individuals reach the PrEP action and initiation stage (Stage 4) when they discuss PrEP with a healthcare provider and get a prescription [76]. In the PrEP maintenance stage (Stage 5), individuals consistently adhere to PrEP and follow guidelines with regular HIV and STI testing [76]. Each step along the continuum serves as a benchmark to evaluate and identify barriers to successful PrEP initiation and maintenance. Prior research using iterations of the PrEP care continuum found that research efforts are needed in the initial stages of the PrEP decision-making process [76,78,79,80,81], specifically during the pre-contemplation and contemplation stages. These earlier stages are key to understanding PrEP readiness, as individuals in pre-contemplation may not yet recognize their need for PrEP, while those in contemplation are aware but uncertain about initiating PrEP.

To date, there is a paucity of research focused on examining CBW’s readiness to initiate PrEP. More qualitative research is needed to specify which obstacles are most impactful and influential for readiness to use PrEP among CBW. The purpose of this exploratory qualitative inquiry was to investigate the precursors to PrEP readiness (i.e., facilitators and barriers related to the decision to initiate PrEP use) and the factors that inform PrEP readiness among CBW in Austin/Travis County and Houston/Harris County, Texas. The primary research question was as follows: What are the facilitators and barriers to readiness to adopt PrEP for HIV prevention among CBW in Austin/Travis County and Houston/Harris County, Texas?

## 2. Materials and Methods

**Figure 1:**
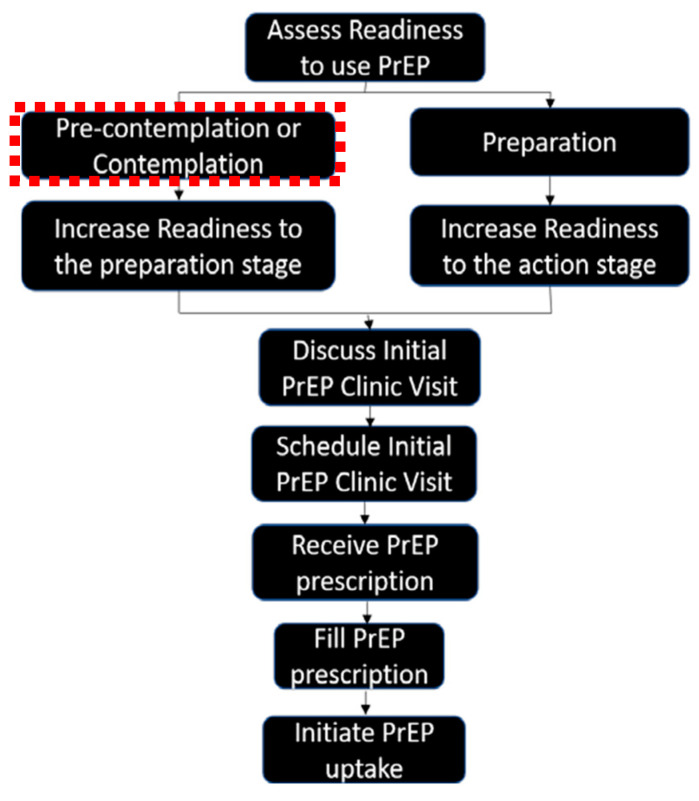
Stepwise approach to content development for the focus group tool used in Aim 1.

Data from the current study are the result of Aim 1 of a larger developmental study. The developmental study had three aims that were planned to be conducted between July 2022 and August 2024. The overarching goal of the developmental study was to create and pilot test a health communication intervention using video-logs to increase readiness for PrEP over time to motivate behavior change, resulting in actionable steps along the PrEP care continuum [75]. The three aims of the study included Aim (1), an observational study design using qualitative methods; Aim (2), intervention development; and Aim (3), a prospective pilot randomized controlled trial of the behavioral intervention using a mixed-method approach. Specific to Aim 1, we report on qualitative findings of examined factors that impact PrEP readiness among a sample of 20 CBW (see Figure 1). The qualitative findings were used to develop health communication materials from the perspective of CBW and inform the content of the video-log-based intervention [82,83,84].

### 2.1. Study Design

All procedures were approved by the institutional review board at the University of Texas Medical Branch (24-0383), UTHealth Houston (HSC-MS-22-0565), and the University of Texas at Austin (STUDY00003981). The study team recruited a purposive sample of 20 PrEP-eligible CBW over a three-month period (March 2023–June 2023) to participate in a virtual focus group. All enrolled participants who completed the focus group received a $50 USD gift card through USPS mail as a thank you for their time.

### 2.2. Community Advisory Board

A community-engaged research (CER) strategy has become a prominent and executive strategy in exploring and evaluating HIV-related interventions among Black communities [85]. Research teams frequently establish and integrate Community Advisory Boards (CABs) to ensure the inclusion of community perspectives, historical and contemporary living contexts, and to prioritize concerns in the development of research protocols, agendas, and implementation strategies. CAB members serve as vital intermediaries, bridging cultural differences and fostering trust by engaging in communication with community members [86], ensuring that community voices, priorities, and concerns are integrated into the research.

Adhering to CER principles when implementing CABs [86], the current study incorporated two site-specific CABs (Houston—Harris County, Austin—Travis County). Individuals were eligible to be a CAB member if they had one or more of the following criteria: (a) community members who had a physical presence within Austin and/or Houston, Texas; (b) had a patient/service population that included CBW; (c) were recognized as trusted sources of information on HIV, reasons for HIV vulnerability, or PrEP information within their respective communities; and/or (d) led or advocated for efforts to improve PrEP access and/or initiation among CBW. In total, each CAB was comprised of four CBW who were reputable community change agents with expertise in public health, medicine, HIV prevention, and/or social work.

During the study period, each CAB and their respective CAB members attended 5 meetings virtually and provided insights and recommendations across critical phases of the study. The CABs assisted with focus group guide development, participant recruitment through their networks, the data analysis process, and the interpretation of the study findings. Each meeting focused on the following:

- Meeting 1: Introductory session encompassing study overview, CAB expectations, and a question-and-answer period;
- Meeting 2: CAB provided feedback regarding the focus group guide and recruitment protocol, ensuring the relevance and appropriateness of inquiry, along with facilitating outreach for recruitment through their established community networks;
- Meeting 3: CAB members engaged in discussions about the initial qualitative findings;
- Meeting 4: CAB members provided additional feedback on preliminary qualitative findings, including emerging themes and their implications;
- Meeting 5: CAB presented the final study results. Meeting focused on discussion of the final results and their meaning and outlining subsequent steps.

Each session lasted up to 70 min. Participating CAB members received $200 USD per CAB meeting as a thank you for their time, amounting to $1000 USD per CAB member for the entirety of their study involvement. Through their expertise and community engagement, the two CABs of this project ensured that the study was inclusive, culturally competent, and community-driven. Their involvement not only improved participant recruitment and data interpretation but also strengthened the collaboration between the researchers of this project and the communities of CBW they aimed to serve.

### 2.3. Recruitment and Enrollment Procedures

All study recruitment and enrollment procedures were site-specific, such that if a participant was enrolled in Harris County, they engaged in the virtual focus group with other participants who also resided in Harris County, and their focus group was led by a research coordinator (RC) in Harris County.

In collaboration with UTHealth Houston and the University of Texas at Austin, a purposive sample of 20 PrEP-eligible Black women in Harris County of Houston, TX, and Travis County of Austin, TX, was recruited through e-flyer distribution through the social media networks of academic and community partners. Each community partner who collaborated with the study committed to distributing e-flyers twice a week during the recruitment period across their social media pages. In addition, they used a purposive snowball sampling technique, which included in-person recruitment and referral strategies through their professional and social networks. All recruitment materials contained information about the study, including the incentive and a method (e.g., QR code, direct weblink) to access the eligibility screener. Prospective participants who viewed the e-flyer were directed to the study’s landing page and prompted to complete an eligibility screening survey via Qualtrics (https://www.qualtrics.com). Eligible individuals included those who self-reported the following: being a cisgender woman, identifying as Black or African American, ≥18 years of age, having had condomless vaginal sex with a cisgender man within the past 12 months, having an HIV-negative serostatus, no emotional or mental discomfort that would prohibit them from participating in the study, fluent in English, had a smart phone or internet access, and were PrEP-naïve (i.e., self-reported never having used PrEP or receiving a PrEP prescription).

The same enrollment procedures were used across both sites. If eligible, individuals were prompted to provide their contact information (i.e., name, email address, phone number) to schedule their focus group session and to be provided additional information about the study. Next, the RC followed up with eligible individuals via phone and/or email to provide a summary of the study purpose and objectives, share the focus group expectations, and discuss the consent process. If the individual agreed to participate in the study, they were sent an email with a link for them to provide informed consent electronically using DocuSign. Once signed, eligible individuals were immediately directed to an online scheduling system to determine focus group session calendar availability. Once scheduled, the participant received an email with the link to the Cisco WebEx software Version 43.6 in which the virtual focus group sessions were conducted, along with the date/time of the session and the name and contact number of the RC who would facilitate the focus group session. The email also contained a link to a pre-assessment Qualtrics survey (Qualtrics, Provo, UT, USA) to collect demographic information, which was completed prior to the scheduled virtual focus group session.

### 2.4. Theoretical Framework

In the larger developmental study from which these data come, a three-pronged theoretical approach was used to guide the study: Wellness Motivation Theory (WMT) [87,88,89], Anderson’s Model of Healthcare Utilization (MHU) [90,91,92], and the Transtheoretical Model of Change (TTM) [77,93,94,95,96]. The current study utilized TTM, an integrative theoretical framework that assesses an individual’s readiness to act on a new, healthier behavior and provides strategies or processes of change to guide an individual through five stages of change: pre-contemplation, contemplation, preparation, action, and maintenance [77,93,94,95,96]. TTM has been successfully used to guide HIV prevention intervention development for use among Black women [97,98,99,100]. In the current study, the findings are representative factors related to PrEP readiness as identified in the pre-contemplation stage of TTM.

### 2.5. Focus Group Guide

The semi-structured focus group guide was adapted from an established focus group guide (FGG) [101] used in a study that evaluated factors related to PrEP initiation among CBW in Texas. For the current study, this adapted FGG was used to discover factors that either facilitated or served as a barrier to PrEP readiness. The content of the FGG included open-ended questions that explored participant concerns about contracting HIV, reasons for getting an HIV test, perceptions of HIV vulnerability as an individual and as a Black woman, perceptions of partners’ HIV risk, PrEP-related knowledge including perceptions of who PrEP is for (self and/or others) and PrEP modalities, concerns about PrEP side effects, perceived pros and cons associated with PrEP use, and reasons for choosing to use or not use PrEP. Open-ended questions collectively guided participants through reflecting on their readiness, barriers and facilitators related to their potential PrEP use. Study participants were also encouraged to describe their personal experiences and the known experiences of their peers on these discussion topics. The current study presents qualitative findings specific to factors that either facilitated or served as a barrier to potential PrEP use, indicating whether participants are ‘ready’ to initiate PrEP (i.e., the pre-contemplation stage of TTM).

### 2.6. Enrolled Study Sample

**Figure 2:**
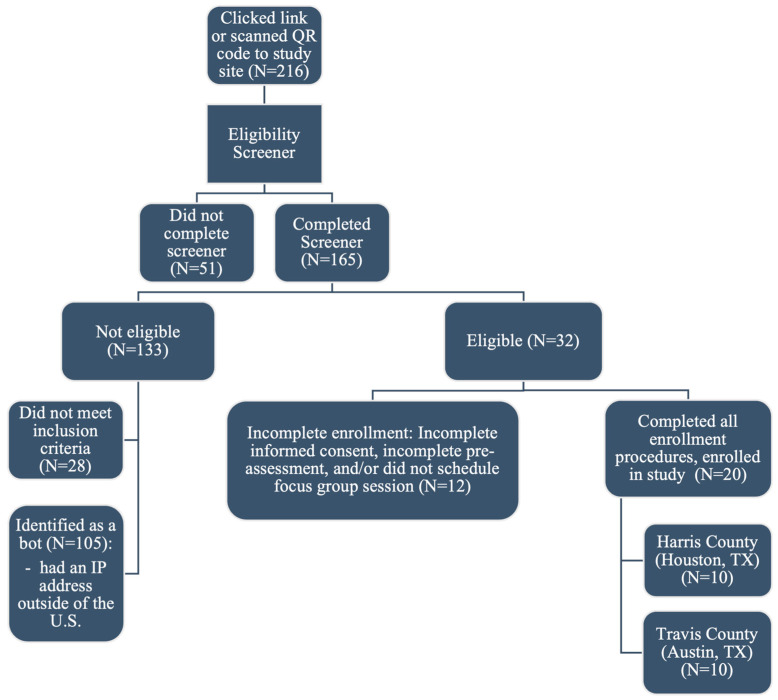
Enrollment of study sample.

The screening tool garnered a total of 216 entries across both study sites. Of the 216 entries, 165 entries had complete screener data. After examining the 165 completed screener entries, 105 (63.4%) were identified as bots (automated programs used to engage in social media and behave in an either partially or fully autonomous fashion and are often designed to mimic human users) and deemed ineligible. Bots were identified through manual checks of the eligibility screener for IP addresses originating outside of the United States and email addresses that showed signs of being illegitimate. These included email addresses containing nonstandard characters or symbols uncommon in the United States (e.g., Â), patterns such as altered combinations of letters and numbers (e.g., a2s3f4g5@gmail.com), or those using unreal or nonstandard email domains (examplefake.biz). Among the 60 remaining screener entries, 28 did not meet the inclusion criteria and were marked as ineligible. The RC contacted the remaining 32 eligible individuals to provide them with more information about the study. Of those contacted, only 20 individuals completed all qualifying actions required to be deemed an enrolled study participant, including providing informed consent, completing the pre-assessment, and scheduling, attending, and participating in a pre-scheduled focus group session. Of the 20 CBW included in the final sample, 10 CBW were from Harris County of Houston, TX, and 10 CBW were from Travis County of Austin, TX (see Figure 2 and Appendix A Table A1).

In three cases at the Houston study site, focus group sessions were scheduled with five consented and enrolled participants. At the onset of these three sessions, only one participant was present within the first 10 min of the session. To respect the time and availability of the enrolled participants who followed through with their commitment to participate in the focus group, the focus group facilitator, with permission from the principal investigator, transitioned to individual key informant interviews. The focus group guide was used as an interview tool to maintain consistency across the study’s methodology. This approach ensured that all participants, whether in focus groups or individual interviews, were asked the same questions, maintaining uniformity in data collection across both formats. In total, five focus groups (4–5 participants per focus group) and three key informant interviews were conducted with PrEP-naïve CBW who lived in Houston (focus groups: N = 2; interviews: N = 3) or Austin, TX (focus groups: N = 3).

### 2.7. Data Analysis

Each focus group and interview discussion was audio recorded, transcribed using transcription services offered through the University of Texas Austin, and lasted approximately 60 min. The RC cleaned the transcripts, ensuring they were de-identified and that the transcribed text was labeled according to the speaker (i.e., the facilitator or the participant). Thematic content analysis was used to analyze the data. The team lead has extensive experience analyzing qualitative data using thematic content analysis [102,103,104,105,106,107].

A three-member coding team co-created an initial codebook in Microsoft Excel using one transcript during an in-person meeting in Austin, TX, USA. All coders used NVIVO 14 software to facilitate the coding of the qualitative data collected. Once the preliminary codebook was developed, each coder independently coded transcripts. Then, coders came together when independent coding was complete, compared codes, discussed differences, and reached a consensus using a constant comparative analytic approach to revise the final codebook [108]. New codes were added only when new codes emerged. Any addition of new codes was reviewed by the team, and duplicate codes were collapsed. All coded data were analyzed for emergent categories and themes [109], and differences were discussed until a consensus was reached. The full thematic analysis of all transcripts resulted in 1872 codes.

Using the statistical software package IBM SPSS v 26.0, the team calculated a Kappa statistic to assess inter-rater agreement between coders. The inter-rater reliability across 1872 codes resulted in a Kappa statistic of −0.227, SE = 0.004 between coder 1 and coder 2, a Kappa of −0.176, SE = 0.004 between coder 1 and coder 3, and a Kappa of −0.183, SE = 0.004 between coder 2 and coder 3. The negative Kappa statistic across all three coders suggests no agreement. However, to improve the validity of the findings, the analysis team generated a report and conducted member checking with the CABs and separately with community partners during professional meetings nationally, roundtable discussions, and presentations of preliminary data and concepts across the local/regional sexual health community. By conducting member checking with the CABs and engaging with community partners through various professional platforms, the research team ensured that the interpretations resonated with both the lived experiences of the target population and the broader sexual health community. This approach also allowed for the validation of initial interpretations against community perspectives, the incorporation of diverse viewpoints into the analysis, and the identification of any potential gaps or misinterpretations in the study’s findings. The feedback received through these channels provided valuable insights that refined the overall understanding and improved the quality and relevance of study findings. By incorporating member checking within the validation process, the team significantly enhanced the credibility and trustworthiness of the study findings.

## 3. Results

Among the 20 CBW enrolled in the study, most were between ages 18 and 29 (n = 16, 80.0%). Equipoise in recruitment, in alignment with the study design, was reached, with half of the participants living in Harris County—Houston, TX, USA (n = 10, 50.0%) and the other half living in Travis County—Austin, TX, USA (n = 10, 50.0%) (see Appendix A Table A1).

All focus group transcripts were coded for two primary themes: facilitators and barriers of PrEP readiness among CBW. A total of 21 themes describing facilitators and barriers to PrEP readiness were identified (see Appendix A Table A2 and Table A3). Each theme was coded for key explanatory ideas that further developed an understanding of participant perspectives of the major facilitators and barriers to PrEP readiness.

### 3.1. Facilitators of PrEP Readiness

Utilizing the TTM, we identified aspects of the pre-contemplation stage that characterize readiness (and unreadiness) to initiate PrEP among CBW in our sample. Six primary themes were identified as facilitators of PrEP readiness: (1) high perceived vulnerability; (2) preferences to ensure continuity of PrEP care; (3) preferred modality; (4) prioritizing PrEP as self-care; (5) exposure to PrEP-related health communication; and (6) preferences for engagement in PrEP. All primary themes and codes of PrEP readiness facilitators are provided in Table 1. Themes that had codes endorsed with the highest frequencies (≥25) are further described.

#### 3.1.1. High Perceived Vulnerability

*Partner exposing participant to HIV vulnerability*. When participants were asked about their belief of whether they could contract HIV, participants perceived that their vulnerability to acquiring HIV comes from one’s partner(s) (*frequency of codes*, *fc* = 58). Responses indicated an awareness that their belief about whether or not they could contract HIV could be inextricably linked to the sexual decision-making of their sexual partner(s).

*People do things and you don’t expect them to do those things. So, I just like to be as careful as I can be on my end*.

(Focus Group 2, Austin, TX, USA)

*I wouldn’t put it past my partners to be at risk of having HIV because I believe if you’re sexually active, you’re always at a risk of contracting HIV*.

(Focus Group 3, Austin, TX, USA)

*So, creating that relationship and creating that comfort level does not eliminate the fact that there is always a possibility of others making different choices or like the prior participant said, unknowingly exposing you to something based on their sexual history, things of that nature*.

(Focus Group 3, Austin, TX, USA)

*Self-awareness of one’s own HIV vulnerability-inducing behavior.* The second most endorsed code related to high perceived vulnerability was one’s self-awareness of HIV *vulnerability-inducing* behavior (*fc* = 39). Responses regarding a sexual partner’s HIV vulnerability suggested an understanding of HIV transmission routes and a general alignment between perceived and actual vulnerability among those with greater self-awareness. Thus, participants with more accurate self-perception of their vulnerability to acquiring HIV tended to assess themselves relative to their partners’ vulnerability more realistically.

*I do believe I have a reason to be concerned [about contracting HIV]. Well, for one, the statistics that you stated and me being a Black woman, I have a higher chance it seems like, of contracting it. So, I am concerned about it. I’m also concerned about it because I’m sexually active. I only have one partner, but I think that I should… Sorry, I’m trying to think. I think that I should be proactive in my healthcare*.

(Focus Group 3, Houston, TX, USA)

*Concern for acquiring HIV*. The third most endorsed code related to high perceived HIV vulnerability included participant concern for acquiring HIV (*fc* = 31). When asked about whether participants felt that they had a reason to be concerned about contracting HIV, responses indicated a logical understanding of the connection between having unprotected sex and the chance of contracting a sexually transmitted disease, specifically HIV.

*I think we definitely have a reason to be concerned. I was really shocked at hearing those statistics, going to different conferences over the years and going to different health conferences at church and what have you when they give you information about HIV and other STDs [sexually transmitted diseases]. Hearing it now again, it’s very shocking. So, I think that’s definitely a concern as far as contracting the disease, whether we take heed to it or not. Definitely*.

(Focus Group 2, Houston, TX, USA)

In lieu of that concern, participants also described a communal concern due to the societal vulnerability imposed by the social construct of race, which they perceived as a rationale to engage in HIV prevention behaviors.

*I was going to say I agree. I feel like if you’re sexually active in any way, you should have some concerns in contracting it. And I think we as Black women should be a little more proactive when it comes to the preventative medicine, so I think the statistics that you just said were shocking, as well, so I agree*.

(Focus Group 2, Houston, TX, USA)

*Routine HIV testing*. The fourth most endorsed code related to high perceived HIV vulnerability was about HIV testing (*fc* = 29). Participants in this study indicated getting tested for HIV/STIs as part of their routine sexual healthcare.

*But I do my part in getting tested regularly*…

(Focus Group 3, Austin, TX, USA)

*I do make sure that I get tested as regularly as possible*.

(Focus Group 3, Austin, TX, USA)

Participants also reported their healthcare provider as a primary reason for why they decided to get tested.

*So, I literally just did my annual last week and my doctor was like, ‘Oh, well, STI screening comes with this if you want it.’ So, my doctor did just let me know that I could get it done. And then I also am proactive. So, I will schedule my own appointments and now that I actually know the timeframe of when to go, I go in and I schedule it accordingly*.

(Focus Group 2, Austin, TX, USA)

#### 3.1.2. Preferences to Ensure Continuity of PrEP Care

This theme focuses on the factors that would influence CBW’s ability to maintain consistent PrEP use over time. Participants discussed various aspects that would help them adhere to a PrEP regimen, specifically the method of administration and how it fits into their existing healthcare routines. Participants expressed that injectable PrEP could be more convenient for long-term use.

*Preferred modality*. A dialogue ensued around reasons why CBW may or may not take PrEP injections every two months. When asked about how realistic it would be for participants to add a PrEP injection every two months into their schedule, their preferred modality was integral to their (hypothetical) continued PrEP care (*fc* = 164). For example, a participant who preferred injectable PrEP stated the following:

*Yeah, for sure. Because like I said, I get the birth control shot every three months, so it’s just one month behind, and it’d be easier. Even going to the OB-GYN, you could always--Let’s say you need a checkup, you can get your shot right there and then. I feel like that method is easier and you won’t have to worry about if I missed it or if I forget it*.

(Focus Group 1, Houston, TX, USA)

Women who may be accustomed to getting a shot for birth control every three months or other injected medication(s) may perceive injectable PrEP as convenient and thus aligning with their current medical and/or sexual healthcare routine.

#### 3.1.3. Preferred Modality

This theme specifically addresses the participants’ preferences for how PrEP is administered, with a focus on injectable PrEP as a facilitating factor. While the previous theme dealt with continuity of care, this theme explores the reasons why CBW might prefer one PrEP modality over another.

*Injection facilitator*. Participants expressed that possibly receiving PrEP as an injectable medication was a facilitating factor (*fc* = 82). Responses varied as to why they preferred this method over oral PrEP. For some participants, injectable PrEP fit into their current sexual health routine.

*I get the birth control shot every three months, so it’s just one month behind, and it’d be easier. Even going to the OB-GYN, you could always--Let’s say you need a checkup, you can get your shot right there and then. I feel like that method is easier and you won’t have to worry about if I missed it or if I forget it*.

(Interview 1, Houston, TX, USA)

*The injection for me as well. I switched from the birth control pill to the shot for the convenience of it. So, the injection once, twice a month. I mean, no, once every two months. That would just be way more convenient than trying to take a pill everyday*.

(Focus Group 2, Houston, TX, USA)

For others, injectable PrEP was preferred due to the inconvenience of taking multiple pills every day or previous experience with forgetting to take their pills regularly.

*I say the injection because it’s kind of hard keeping up with taking pills, the same way as with birth control*.

(Focus Group 5, Houston, TX, USA)

*I would say injection because, I know for me, even with my birth control pills, sometimes I forget to take them. So, I’d rather just have a shot every two months versus having to remember to take a pill every day*.

(Focus Group 2, Houston, TX, USA)

*Daily pill facilitator*. Likewise, ‘daily pill facilitator’, or using PrEP as an oral daily pill, was among codes that were also highly endorsed (*fc* = 30), although less endorsed than injectable PrEP. Oral PrEP was preferred among participants who believed they could integrate oral PrEP into their existing medication routines, had an aversion to injections, perceived difficulty in consistently scheduling and attending a bi-monthly medical appointment, or simply preferred a pill-based medication over injectable options.

*The women in my family, if they are already taking a medication, it’d probably be easier for them to remember to take that or if they don’t like needles, so they probably want to do the pill*.

(Focus Group 1, Austin, TX, USA)

*I would say the pill just because I don’t like shots. I’m not a shot type of person and my schedule is crazy and hectic, so I don’t want to go to the doctor. So, the pill would be best*.

(Focus Group 2, Houston, TX, USA)

#### 3.1.4. PrEP as Self-Care

*Prioritize PrEP as self-care*. When the interviewer asked if PrEP can be a form of self-love and empowerment, prioritizing PrEP as self-care was a key concept mentioned by participants (*fc* = 107).

*I guess taking PrEP and doing all these other things would be a form of self-love to your person, to yourself, or whatnot. And even for others too, because you’re taking these steps to make sure that you’re good and other people, the people that you’re dealing with around you are good*.

(Focus Group 3, Austin, TX, USA)

*I would say as far as making decisions to take PrEP could be a form of self-care. Just to step in a good direction if you feel like it is something that you do need. It allows you to provide also grace for yourself because if being sexually active is something that you want to do for yourself, you’re allowed to and also have those precautions as mentioned earlier*.

(Focus Group 1, Austin, TX, USA)

Another participant stated the following:

*Well, it’s just freedom from the stigma. You know, don’t have to have that stigma on you. You don’t have to worry about catching something that you can’t get rid of and you know, you can love yourself more. Just knowing that you taking care of yourself and you doing what you prepping. You know what I’m saying? You doing what you need to do. So just take care of yourself and the others around you*.

(Interview 3, Houston, TX, USA)

#### 3.1.5. Exposure to PrEP-Related Health Communication

*Exposure to PrEP-related media*. Participants were asked about their impression of advertisements (ads) seen in commercials. Some participants reported being exposed to PrEP-related health communication (*fc* = 67) through radio and TV ads/commercials. Ads distributed through these media outlets were described as being inclusive by depicting individuals who used PrEP as a part of daily living. Participants also noted the lack of representation of CBW within these PrEP-related ads and suggested that they should be more inclusive of CBW in order to better appeal to this population.

*But if they actually included us [Black women] within it [ads], then I think we would be more inclined to be like, ‘Hmm, well what’s that? And let me learn a little more about that.’*

(Focus Group 2, Austin, TX, USA)

#### 3.1.6. Preferences for Engagement in PrEP

*Provider recommendation for health-related choices*. During each qualitative session, the facilitator shared information about PrEP side effects and asked participants for their thoughts concerning the benefits of PrEP versus the side effects. Provider recommendation was the most endorsed code related to participant preferences for engagement in PrEP (*fc* = 38). Responses suggested that if PrEP was recommended by their provider, they would be more likely to consider using it. Some participants described the side effects of PrEP as commonplace. One participant stated the following:

*I mean, if it [PrEP] can prevent you from getting HIV, it’s a… It is just one of those things. Like I said before today, I never heard of it. Like I said, if I spoke with my doctor and there was something that she told me that I needed to do or something like that, I probably would be more prone to do it regardless of what the side effects are. Because those side effects that you named are basic side effects, like you said, that we can get from anything. So, I would be more prone to do it coming from a specialist*.

(Focus Group 5, Houston, TX, USA)

### 3.2. Barriers to PrEP Readiness

The study team identified six primary themes related to barriers to PrEP readiness: (1) concerns with modality; (2) concerns with continuity of PrEP care; (3) low perceived vulnerability; (4) PrEP stigma; (5) aversion to taking manufactured medication; and (6) lack of PrEP knowledge as a barrier. Table 2 lists all themes related to barriers and their accompanying codes. Themes with codes endorsed with the highest frequencies (≥25) are further described.

#### 3.2.1. Concerns with Modality

To gauge participant’s interest in other ways PrEP could be administered, participants were asked about their thoughts on using PrEP if it was administered as a monthly pill, at a pharmacy as a self-injection, or available as a vaginal ring. The choice of PrEP modality and its method of administration significantly influenced an individual’s readiness to adopt PrEP as an HIV prevention strategy.

*Pill barriers*. When the facilitator asked what would make the participants feel the most comfortable or confident with taking PrEP as either a pill or injection, barriers related to oral PrEP emerged (*fc* = 57). One participant echoed the sentiment of a fellow participant who said that she is horrible at taking her birth control pills on time, stating the following: ‘*I know myself. I’m not a timely person at all. I’m also on birth control. I’ve never taken the pill*.’ Individuals who lack the ability to adhere to an oral pill regimen for other prevention or treatment care options may not be ready to use PrEP as an oral pill option.

*Barrier to vaginal ring PrEP as future option*. Concerns with PrEP modality also included whether participants viewed vaginal PrEP as a possible option for HIV prevention. When asked about using PrEP as a vaginal ring, participants reported disinterest due to either being uncomfortable with this form of PrEP or fear related to the way it must be inserted for use.

*Because I know my friends who do take the birth control like the NuvaRing. I never thought of that as an option for me because I don’t know, I feel uncomfortable doing it*.

(Focus Group 3, Austin, TX, USA)

*…the vaginal ring, I personally would not want that just because I’ll be thinking, ‘Oh, well what if it slipped out or something like that.’ And I wouldn’t want that concern*.

(Focus Group 3, Austin, TX, USA)

*I wouldn’t want anything [PrEP vaginal ring] foreign in my body that does not have to be there, I guess. I know it’s a preventative method, but I wouldn’t want to do it*.

(Focus Group 5, Houston, TX, USA)

*Injection barriers*. With respect to injectable PrEP, participants voiced concerns about the injection site and whether it would be painful. Injectable PrEP may not be a viable option for participants with an aversion to needles and low pain tolerance.

*Or with the injection, maybe pain in the injection area*.

(Focus Group 3, Austin, TX, USA)

*But with the injection, I just thought about the injection site. I don’t know where exactly it’s being injected at. So I don’t know, that could be a concern to me*.

(Focus Group 3, Austin, TX, USA)

Costs associated with injectable PrEP was also a voiced concern among study participants.

*With the injections, for me, it’s all about cost. What if I didn’t have health insurance or anything like that? If I needed to get it every two months, can it be covered consistently and the cost about that*.

(Focus Group 3, Austin, TX, USA)

*Barrier to self-injection PrEP as future option*. When asked about their thoughts about using PrEP if it was administered as a self-injection, participants were concerned about injecting themselves with PrEP (*fc* = 27), as some participants were unfamiliar with self-injections or expressed an aversion to needles in general.

*And then with the shot, I also have concerns with that because first of all, me administering a shot to myself without being trained and whatnot, I feel like there’s just so many things that could go wrong, and it could break and get stuck in my skin, this and that. There’s just so many factors*.

(Focus Group 3, Austin, TX, USA)

*The self-injection, I feel like I personally can take needles well, but I wouldn’t want to have that responsibility. Even though it’s on myself, for myself, I would want a healthcare professional who has had the experience to inject that in me*.

(Focus Group 3, Austin, TX, USA)

#### 3.2.2. Concerns Regarding Continuity of PrEP Care

*Concerns with PrEP contraindications or side effects*. The facilitator also inquired about reasons that would make participants hesitant to take PrEP medication as a pill or injection. With respect to the theme ‘Concerns regarding continuity of PrEP care’, concerns with PrEP contraindications and side effects was identified as a barrier (*fc* = 35) due to the expressed concerns about potential long-term side effects of PrEP and its impact on one’s overall health. Participants also voiced their uncertainty about the medication’s bodily effects.

*Not knowing the side effects of it, how long has this been an option, what are the side effects long term? Because I’m older*.

(Focus Group 5, Houston, TX, USA)

*I mean, of course I would not want to be sick. I would not want to have weight gain, I wouldn’t want something that’s going to cause other issues going on with me, because I’m taking this. Those would be major things, especially the being sick part. I feel like sometimes, any kind of thing you put in your body can change your body, because it’s not natural*.

(Interview, Houston, TX, USA)

#### 3.2.3. Low Perceived HIV Vulnerability

*Low perceived HIV vulnerability*. Women were asked to describe reasons other women would choose not to take PrEP for HIV prevention. Low perceived vulnerability of acquiring HIV was the most endorsed code across participants (*fc* = 50), further indicating that self-perceived vulnerability is an important indicator of PrEP readiness.

*Yeah [I think my chances are very low for getting HIV]*.

(Interview, Houston, TX, USA)

*Or they just feel like they’re the exception to the rule on some type of sense, which we all know that’s not the case, but maybe there’s some women that really feel like it really doesn’t apply to me, so it’s not something I would want to take*.

(Focus Group 4, Houston, TX, USA)

*Certainty that PrEP is not an option*. The second most endorsed code related to low perceived HIV vulnerability was participant certainty that PrEP is not an option for them (*fc* = 28). Some participants outright rejected PrEP as a viable HIV prevention option without a specific reason, despite understanding that they were eligible for PrEP. The quoted text from participants where this code was used reflected a self-awareness of personal choice and a certainty in decision-making regarding a clear stance that PrEP was not the preferred option for HIV prevention in their lives.

*I just personally wouldn’t [take PrEP], too*.

(Focus Group 2, Houston, TX, USA)

*But I don’t think I would take PrEP at all because it’s just like I don’t think I would try to put myself in… I would ask your status or something*.

(Focus Group 2, Houston, TX, USA)

#### 3.2.4. PrEP Stigma

Participants in this study expressed concerns about PrEP disclosure, assumptions of promiscuity, judgment about sexual behavior, and misunderstanding of who PrEP is intended for due to using PrEP.

*PrEP stigma*. Participant’s apprehension toward PrEP use was due to potential judgment (i.e., anticipated stigma) from family, friends, or relationship partner(s) if they decided to use PrEP.

*I mean, I feel like people are just… They can be just judgy by nature, so I feel like you may still get some looks or what are you really doing? I feel like that’s just going to come*.

(Focus Group 2, Houston, TX, USA)

Participants also believed there would be an assumption about their own HIV status (i.e., having an HIV-positive status) due to misinformation and misconceptions about who PrEP is intended for if they chose to use PrEP for HIV prevention.

*I think they might probably judge a little bit, but I think they would just assume, you know what I’m saying? Because when I first heard it, I just made an assumption, but I think some people might make an assumption like, oh you have it already. But no, that’s just to prevent it*.

(Interview, Houston, TX, USA)

*I think that would be negative in a way, just based off of, like I said earlier, the presumption that you may have something, and that’s just because people don’t know or people don’t understand. And no matter how much you try to explain something to somebody, they probably just don’t get that. ‘Well, why would you still take that if you don’t have it?’ That’s the mindset*.

(Interview, Houston, TX, USA)

*Perceptions of high vulnerability with PrEP use*. Some participants believed that disclosing their PrEP use would lead others to assume they were engaging in behaviors that would increase their vulnerability to HIV, such as having multiple partners or engaging in condomless sex or that one’s sexual partner is HIV-positive.

*I would say that people would know that we were sexually active. If we vocalize that like, ‘Oh, yeah. We use PrEP, and da, da, da, da.’ It would make people aware that we’re sexually active. And if we said that we use PrEP, it would also… Maybe the first thing that comes to somebody’s mind is that you’re not only sexually active, but with multiple people that’s why you feel the need to have HIV PrEP*.

(Focus Group 3, Austin, TX, USA)

*I feel like for, I guess my family, or probably, if I told my sister, I guess she would believe or she would think like, ‘Oh, are you sleeping with somebody who’s already infected with HIV?’ Because I feel like that’s one of the options when you are engaging with somebody who is HIV-positive*.

(Focus Group 3, Austin, TX, USA)

#### 3.2.5. Aversion to Taking Manufactured Medication

*Concerns with PrEP contraindications or side effects*. In relation to the theme ‘Aversion to taking manufactured medication,’ the code ‘concerns with PrEP contraindications or side effects’ was identified. However, within this theme, the code relates to concerns about taking PrEP medication regarding the possibility of medication interactions.

*Well, there’s not really any information out there at least that’s geared toward women about what the side effects are or the potential ramifications of taking it. You don’t know if you have to be screened for allergies. You don’t know if you can’t have the PrEP shot or the oral if you have certain preexisting health conditions. There’s just not a lot of information. So it’s taking a shot in the dark really*.

(Focus Group 2, Austin, TX, USA)

Participant responses also emphasized that PrEP hesitancy is linked to the medication instead of the modality by which the medication is administered.

*Certainty that PrEP is not an option*. PrEP was not perceived as a needed HIV prevention option for some participants in this study (*fc* = 28). Participants expressed a general reluctance to medications unless absolutely necessary and preferred to do research before considering any new medication.

*I agree as far as I’m quick to say no [to taking PrEP] also because it’s hard for me to trust things or programs or systems automatically without just seeing what comes out of it myself or not myself, but through other experiences or testimonies themselves*.

(Focus Group 1, Austin, TX, USA)

*Yeah, it’s a no for me. It’s not just PrEP. It would be a no for me with a lot of medication. That’s just me*.

(Focus Group 1, Houston, TX, USA)

#### 3.2.6. Lack of PrEP Knowledge as a Barrier

*Lack of PrEP knowledge as a barrier*. In addition to the PrEP modalities discussed with participants (oral and injectable PrEP), the facilitators also informed participants of additional PrEP modalities currently under clinical trials or under consideration, such as a vaginal ring, a monthly pill, and a self-injection at a pharmacy. Participants were asked if their readiness for PrEP would change if there were other options (like these) for PrEP. Participants expressed that a lack of knowledge of PrEP was a barrier (*fc* = 60), limiting their ability to conceptualize how other PrEP modalities could be applicable to them. Participant 3 in Focus Group 2 in Austin, TX, USA, described inequities in the shared decision-making between her and her healthcare provider versus the experience of her gay male friend and their healthcare provider. She asserted that the exclusion of women in the marketing is misleading, which is reinforced when healthcare providers do not engage women in conversations about PrEP to increase their knowledge and ability to make informed sexual health decisions.

*I wish there was just more information or just the same way… Because I have a friend who is a gay man and he gets those conversations had with him, but when I go to the doctor, I don’t get those conversations. So, it’s being not equal, but just basically if you’re going to tell one person, you need to tell all people about their options because again, it’s marketed as if women aren’t allowed to take it. So, it’s very misleading. So, it’s also making sure doctors are knowledgeable in sharing that information so that everyone can be protected and careful and things like that, because people are going to do what they’re going to do. So just knowing what your options are and how you can help yourself is really important*.

(Focus Group 2, Austin, TX, USA)

## 4. Discussion

Identifying plausible pathways to increase PrEP initiation among CBW is a necessary action if we are to realize the goals of the Ending the HIV Epidemic plan by 2030. Before we can effectively increase the initiation of PrEP—an HIV prevention modality that has been publicly available since 2012—researchers, interventionists, as well as population and public health practitioners must discover less obvious reasons why CBW are not initiating PrEP use. Given the paucity of literature describing precursors for PrEP readiness among populations with significant vulnerability to HIV and low rates of PrEP uptake, research is needed to fill this gap.

To address this research gap, the present study examined the facilitators and barriers related to PrEP readiness within a sample of CBW. Utilizing the TTM, we identified culturally specific aspects of the pre-contemplation stage that characterize readiness (and unreadiness) for PrEP use among CBW. Study findings revealed several key factors that influence PrEP readiness among this population: perceived vulnerability for acquiring HIV, lack of PrEP knowledge, PrEP messaging within media outlets, PrEP modality (oral versus injectable PrEP), concerns with PrEP side effects, medical mistrust, and PrEP-related stigma. These findings are a significant, novel contribution to the existing literature on this topic, which has acknowledged but must still examine the causes of CBW’s unwillingness to utilize PrEP. Gaining a better understanding of the predictors of PrEP readiness enables the sexual health field to meet HIV vulnerable populations where they are and establish solutions that best align with CBW’s capacity and ability to adopt effective prevention options.

### 4.1. Perceptions of Personal HIV Vulnerability

Understanding CBW’s beliefs about their personal vulnerability to acquiring HIV is a vital factor in accurately gauging one’s motivation to either engage in protective behaviors or partake in activities that maintain or exacerbate current HIV-related vulnerabilities [32]. In this study, CBW with a high perception of their personal HIV vulnerability understood that their HIV vulnerability was associated with their current and historical behaviors of engaging in condomless sex and/or maintaining multiple sex partners. These women also expressed a self-awareness whereby a lack of knowledge concerning their partner’s other sexual activities likely increased their personal vulnerability to HIV. This vantage point was described often by participants, indicating that study participants may have realistic concerns about acquiring HIV. This finding challenges decades of literature whereby CBW have been reported to have low perceptions of HIV vulnerability [40,43,53,110,111,112,113,114,115]. Our findings highlight a significant shift in how CBW perceive their HIV vulnerability, suggesting a more nuanced understanding of risk than previously reported. This self-awareness reflects a more proactive recognition of risk, challenging longstanding narratives that portray CBW as underestimating their HIV susceptibility. These insights suggest that CBW may be better equipped to assess their risk than earlier research implied, underscoring the importance of interventions and healthcare practice that builds on this awareness to promote PrEP use and other HIV preventive behaviors in this population. A more recent study among CBW found that disillusionment with low perceptions of HIV vulnerability was hypothesized to be linked to cognitive dissonance [116,117]. As a result of the equipoise between actual and perceived perception of reasons for HIV vulnerability within this study cohort, this group of participants may be more likely to take a proactive approach to their sexual healthcare with consistent engagement in routine HIV/STI testing and possible PrEP initiation once readiness is established. Prior studies have also shown that Black women who are PrEP aware but have low perceived vulnerability of acquiring HIV are less likely to use PrEP [64,118,119], while those with medium to high perceived HIV vulnerability are more interested in using PrEP [32,120]. It is clear that understanding CBW’s perceptions of their personal HIV vulnerability is a vital part of the paradigm for enhancing their PrEP readiness.

To effectively increase CBW’s perceptions of their personal HIV vulnerability and promote PrEP readiness, culturally tailored education programs are needed. Progress is already being made in this area, as the E-WORTH intervention has shown promise in enhancing HIV vulnerability perceptions among Black women [121,122,123].

### 4.2. PrEP Messaging

To effectively increase PrEP readiness and initiation among CBW, increased visibility and enhanced representation of CBW are needed within PrEP messaging and PrEP-related media marketing advertisements (ads), specifically among CBW who engage in heterosexual sex. The lack of representation of CBW within TV ads/commercials contributes to a perception that PrEP is not relevant or intended for CBW, thus limiting their PrEP readiness and hindering possible PrEP initiation in this population. To address this issue, it is imperative that PrEP marketing campaigns and public health messaging intentionally include diverse representations of CBW, showcasing them as potential PrEP users. Media marketing strategies must include details about PrEP effectiveness, ease and feasibility of use, accessibility, acceptability, affordability, and safety [64], as well as information about newer PrEP modalities like injectable PrEP [124,125,126]. Furthermore, study findings here indicate that future media strategies may be more successful at increasing PrEP readiness if they frame PrEP use as a self-care behavior rather than solely as an HIV prevention method. This approach can help to communicate PrEP use as a more universal health option and encourage a broader understanding of its role in improving the health of the population, potentially leading CBW to view it as a sexual health tool, which aligns with current Centers for Disease Control and Prevention recommendations for PrEP [20,33,127].

### 4.3. PrEP Modality

Findings from this study are among the first to describe CBW’s perceptions about injectable PrEP. Participants expressed comfort with PrEP as a bi-monthly injection above the daily oral pill based on their general preference (pill versus injection). For instance, participants who preferred injectable PrEP had familiarity with getting an injection (e.g., birth control injections), preferred its convenience, and had a desire to reduce the inconvenience of taking a daily pill. In contrast, those who favored daily oral PrEP reported that they could easily integrate an oral pill into their existing medication routines, expressed an aversion to injections, and found it challenging to attend bi-monthly medical appointments. Nydegger et al. [128] identified similar findings in their longitudinal qualitative study among 18 cisgender Black and Latina women in Travis County, TX. Women in their study sample who were interested in PrEP preferred long-acting injectable formulations due to their convenience and the elimination of daily pill tracking. Given the newness of PrEP as an injectable medication, there is a lack of literature assessing and quantifying notions of PrEP acceptability of injectable PrEP among CBW. However, the findings here infer that more research is needed to further explore this difference in acceptability based on preferred PrEP modality.

### 4.4. Medical Mistrust

Currently, there is a lack of understanding about the specific implications that medical mistrust can have on CBW in relation to PrEP readiness. Our findings suggest that medical mistrust presents a multifaceted barrier to PrEP readiness among CBW [41,54,59]. Similar to prior research [49,52,56,119,129], women in this study expressed mistrust and concerns about the cultural competence and sensitivity of healthcare providers, particularly regarding whether providers are experienced with the administration of PrEP to CBW. CBW’s skepticism about the quality of care they would receive may lead to a reluctance to seek necessary sexual healthcare, further exacerbating feelings of mistrust toward healthcare systems that have historically marginalized Black women’s needs and experiences. Overall, addressing medical mistrust with CBW is necessary to enhance their comfort with following the guidance of the healthcare providers who provide their sexual healthcare, especially regarding HIV prevention and PrEP readiness [64].

### 4.5. PrEP-Related Stigma

Social and peer influence can play a role in PrEP stigma, leading to a reluctance to discuss PrEP with family, relationship partners, and/or healthcare providers. Perpetual fear of being stigmatized can stifle one’s ability and willingness to communicate about PrEP, which can potentially impact the decision to adopt and/or adhere to an effective PrEP regimen. Participants in the current study were apprehensive about using PrEP due to others’ (i.e., immediate family members and the broader Black community) assumptions that women taking PrEP are promiscuous or living with HIV [59,118]. This finding reflects broader societal misconceptions that can contribute to misinformation and disinformation regarding what PrEP is, who it is indicated for, and its potential sexual health benefits. Study findings suggest that some CBW have an aversion to taking manufactured medication based on cultural views of medication within Black communities [118], further complicating the landscape of PrEP readiness for CBW at the communal and societal levels. Similar findings were identified in prior research. For example, Sophus et al. [130] found that CBW with greater expected PrEP disapproval by others were less likely to have plans to use PrEP in the next 3 months. Goparaju et al. [131] found that when family/friends assumed that taking PrEP indicated that an individual was HIV-positive, this influenced whether or not CBW would make the decision to adopt and use PrEP. Moreover, HIV stigma is still strong and prominent within the Black community. Thus, it is not surprising that these same sentiments are reflected toward PrEP, a highly effective HIV prevention medication. 

Countering PrEP-related stigma requires culturally tailored interventions that educate communities about the purpose and benefits of PrEP while dismantling harmful stereotypes about its users. Efforts should focus on normalizing conversations about HIV prevention and promoting PrEP as an empowering tool for sexual health rather than an indicator of risk or disease. Collaborating with community-based organizations and leaders within Black communities can help disseminate PrEP information and address misinformation about HIV vulnerability, PrEP eligibility, and effectiveness in spaces where CBW congregate. These efforts can help to ensure the relevance and accessibility of PrEP-related messaging. Ultimately, by refining these approaches at the communal and societal levels through intervention approaches, the probability of improving PrEP readiness at these population levels will likely increase in tandem.

### 4.6. Policy Implications

The study findings here have several policy implications. First, funding priorities must support community-based participatory research focused on PrEP readiness among CBW. This research is central to the development of culturally responsive interventions that address barriers to PrEP readiness, initiation, and adherence among CBW. Second, recommendations for women’s healthcare must encourage the integration of PrEP counseling into standard sexual and reproductive healthcare for cisgender women. It is also critical that providers working with CBW adopt a shared-decision-making model of care to support CBW in making the best choices for themselves. Providers have a social responsibility to engage CBW with an awareness and cultural sensitivity to the compounded historical trauma of their racial group with the U.S. healthcare system. As such, providers have the opportunity to take the onus and responsibility of initiating and leading sexual health discussions with CBW with an awareness of a collective HIV vulnerability at the societal level that surpasses individual behaviors. Fidelity to such practices requires policies in women’s healthcare settings that support diversity and inclusion in the healthcare workforce. These workplace policies must also encourage providers to overcome stigma and implicit biases contributing to medical mistrust/distrust and other barriers to PrEP use among CBW. Third, policies and research priorities must focus on increasing the inclusion of CBW in PrEP implementation research. This requires funders of clinical research to adopt policies that require adequate sampling and inclusion of CBW. These changes in policy will provide more information about the effectiveness, efficacy, and safety of PrEP for CBW, as well as address potential contraindications between PrEP and other medications commonly used by cisgender women (e.g., birth control). Lastly, funders must increase support for innovative outreach and marketing that reframes PrEP to represent CBW’s communities and priorities, improves PrEP knowledge among CBW, and develops novel community-based distribution models in order to promote PrEP readiness among CBW. Informed by the study findings, Table 3 outlines policy-relevant recommendations for stakeholders across research, healthcare, and industry that would address PrEP readiness among CBW.

### 4.7. Limitations

Study findings must be interpreted within the context of some study limitations. First, the inclusion of a small sample size limits the generalizability of the study findings to referent other CBW who live in other cities in Texas, a study limitation that is similar to other qualitative studies exploring facilitators and barriers to PrEP initiation among Black women [58,132]. A second potential limitation may have stemmed from questions about sexual behaviors throughout the focus group guide that could be perceived as sensitive and personal in nature, which may have led to social desirability bias. Consequently, participants may have understated some responses due to concerns of stigma surrounding sexual behaviors to align with perceived social norms. To minimize the impact of this potential bias, the focus group facilitator established a welcoming and non-judgmental atmosphere, validated all participants’ contributions to the conversation, and encouraged anonymity and confidentiality, allowing participants to keep their videos off if desired and requiring they change their screen name. Although allowed to keep their videos off, the majority of participants kept their cameras on and actively engaged in each session. Third, more detailed information about injectable PrEP was not uniformly provided, specifically in regard to the clinical nature of the injection as an intramuscular injection. This may have impacted the way participants viewed this PrEP modality as a viable option, given that some participants expressed fears about injectable PrEP and whether the injection hurts.

Additionally, PrEP is maximally effective for receptive vaginal sex after 21 days of daily use compared to only 7 days of daily use for receptive anal sex [133]. Although recent engagement in heterosexual sex was a part of the inclusion criteria for the current study, the data collection tools used did not capture the type of sex had by the participants, nor how often they engaged in those sex behaviors. If collected, this information could have better informed us of participants’ specific HIV vulnerability levels, the appropriateness of the type of PrEP as a prevention method given their sexual practices, and the potential adherence challenges they might face based on the type and frequency of their sexual activities. Future research will include qualitative methods that contain questions about sexual behaviors and preferences among study participants to identify the factors that were missing in this initial assessment of PrEP readiness among a sample of CBW in Houston and Austin, TX, USA, two priority EHE jurisdictions in Texas. Also, while medical mistrust was identified as an indicator of PrEP unreadiness, the focus group questions did not explicitly probe for medical distrust. Future research should consider intentionally probing for evidence of medical distrust, ensuring that questions are non-leading, as this could provide an accurate understanding of its role in PrEP readiness compared to medical mistrust.

## 5. Conclusions

The persistent and disproportionate rates of new HIV among CBW compared to women in other racial groups underscore the need for targeted interventions to address this pressing public health challenge. Inequities in HIV acquisition and PrEP initiation rates are perpetuated by a myriad of factors, including stigma, mistrust/distrust of healthcare providers, lack of representation of Black women in PrEP-related media, and structural barriers to accessing healthcare. Efforts to normalize PrEP usage and increase access must address these multifaceted challenges and be tailored to the specific needs and experiences of CBW. By understanding and addressing the factors that inform PrEP readiness, we can effectively help Black women feel empowered to make informed decisions about their sexual health, ultimately mitigating the disproportionate impact of HIV within this demographic while contributing to the advancement of sexual health equity for Black women at the population level.

## Figures and Tables

**Table 1:** Primary themes and codes of facilitators to PrEP readiness with cisgender Black women identified through thematic analysis of focus group data from Houston and Austin, TX.

Theme	Codes	Frequency of Codes
**High perceived vulnerability**	***10 codes***	**204**
	Partner exposing participant to HIV vulnerability	58 **
	Self-awareness of one’s own HIV vulnerability behavior	39 *
	Concern for acquiring HIV	31 *
	Routine HIV testing	29 *
	Current engagement in HIV vulnerability behaviors	15
	Awareness that PrEP reduces vulnerability of HIV transmission in serodiscordant relationships	8
	Awareness of increased HIV vulnerability as a Black woman	7
	High vulnerability perceived with condomless sex	7
	Awareness of increased HIV vulnerability with multiple sex partners	6
	High perceived severity	4
**Preferences to ensure continuity of PrEP care**	***3 codes***	**182**
	Preferred modality	164 ***
	Ease of PrEP access	11
	Continuity of PrEP care	7
**Preferred modality**	***9 codes***	**176**
	Injection facilitator	82 **
	Daily pill facilitator	30 *
	Monthly pill as future PrEP option	14
	Clinician-administered injection	13
	PrEP patch as future option	13
	Preference for PrEP as a vaginal ring (includes vaginal ring as a future PrEP option, preference for a ring for PrEP, and PrEP as a vaginal ring)	10
	Desire for alternative modalities (includes desire for diversity of PrEP modalities)	6
	Gummy-style PrEP as future option	4
	Liquid PrEP as future option	4
**Prioritizing PrEP as self-care**	***6 codes***	**155**
	Prioritize PrEP as self-care	107 ***
	Sexual autonomy with prevention	21
	Sexual communication with partner	13
	Accountability for cisgender Black women to engage in preventative medicine	6
	Awareness that PrEP can reduce chances of acquiring HIV	6
	PrEP willingness driven by fear	2
**Exposure to PrEP-related health communication**	***5 codes***	**99**
	Exposure to PrEP-related media	67 **
	Testimonials from cisgender Black women who take PrEP	13
	Normalize PrEP messaging through media	8
	Inclusivity in PrEP-related media	8
	Health communication to increase PrEP use and adherence	3
**Preferences for engagement in PrEP**	***6 codes***	**84**
	Provider recommendation for health-related choices	38 *
	Desire for transparency around PrEP (duplicate of ‘need for transparency with pros and cons of PrEP use’)	14
	Desire for OBGYN to discuss PrEP options	12
	Trusted sources inform health-related decisions	12
	Desire for OBGYN to discuss PrEP options with women of color	5
	Community engagement to promote PrEP	3

*Note*. *** frequency of codes ≥ 100; ** frequency of codes ≥ 50; * frequency of codes ≥ 25.

**Table 2:** Primary themes and codes of barriers to PrEP readiness with cisgender Black women identified through thematic analysis of focus group data from Houston and Austin, TX.

Themes	Codes	Frequency of Codes
**Concerns with modality**	***10 codes***	**210**
	Pill barriers	57 **
	Barrier to vaginal ring PrEP as future option	31 *
	Injection barriers	29 *
	Barrier to self-injection PrEP as future option	27 *
	Concern for pill side effects or interactions	22
	Low perceived ability to adhere to daily pill	17
	Concern for injection-related side effects	14
	Barrier to monthly pill as future option	5
	Injection cost	4
	Concern with injection site	4
**Concerns with continuity of PrEP care**	***10 codes***	**118**
	Concerns with PrEP contraindications or side effects	35 *
	Concern for pill side effects or interactions	22
	Burden to continuity of care	18
	Low perceived ability to adhere to daily pill	17
	Cost	9
	Concern with injection site	4
	Injection cost	4
	Distance to clinic	3
	Concern about long-term mental health implications	3
	Presumption that the pill’s way of preventing pregnancy applies to PrEP	3
**Low perceived HIV** **vulnerability**	***7 codes***	**117**
	Low perceived HIV vulnerability	50 **
	Certainty that PrEP is not an option	28 *
	Expectation of monogamy	23
	Low perceived severity	6
	No awareness of HIV burden to cisgender Black women	4
	Confidence in preventative measures outside of PrEP	3
	Choose not to view sexual health as a serious thing	3
**PrEP stigma**	***6 codes***	**97**
	PrEP stigma	49 *
	Perceptions of high vulnerability with PrEP use	27 *
	Family stigma around sex (formerly lack of family support due to stigma)	11
	PrEP unwillingness linked to privacy concerns (formerly PrEP unwillingness linked to lack of independence)	6
	PrEP use should not be linked to careless sexual activity	2
	Peers may assume a PrEP user has HIV	2
**Aversion to taking** **manufactured medication**	***6 codes***	**97**
	Concerns with PrEP contraindications or side effects	35 *
	Certainty that PrEP is not an option	28 *
	Concern for pill side effects or interactions	22
	Preference for holistic approach to health as a PrEP barrier	7
	Concern about long-term mental health implications	3
	Does not equate PrEP use with self-care	2
**Lack of PrEP knowledge as a barrier**	***3 codes***	**73**
	Lack of PrEP knowledge as a barrier	60 **
	Lack of exposure to PrEP media	9
	Lack of PrEP exposure by provider	4

*Note*. ** frequency of codes ≥ 50; * frequency of codes ≥ 25.

**Table 3:** Policy-relevant recommendations by stakeholder audience for improving PrEP readiness among cisgender Black women.

Stakeholders	Recommendations
**Researchers and** **Interventionists**	**Community-Based Research:** Continue to conduct community-based participatory research to understand sociocultural influences on perceived HIV vulnerability and PrEP adoption among cisgender Black women. **Culturally Responsive Interventions:** Develop interventions that integrate cisgender Black women’s lived experiences, including structural barriers and medical mistrust/distrust, and consider how social networks (peers, partners, and community) shape vulnerability perception and PrEP initiation. **Preference Assessments:** Conduct mixed-methods research to understand cisgender Black women’s preferences for different PrEP modalities and factors influencing choice (e.g., lifestyle, convenience, side effects, autonomy concerns) that addresses how social norms, stigma, and partner influence shape preferences for PrEP modalities.
**Healthcare** **Providers**	**Integrate into Routine Women’s Healthcare**: Implement PrEP counseling as a standard component of sexual and reproductive health visits for cisgender women. **Shared Decision-Making Models:** Implement patient-centered counseling that acknowledges the weight of provider recommendations and potential for provider bias and presents all available PrEP modalities to support cisgender Black women in choosing the best option for their needs. **Implicit Bias Training:** Train providers to engage in non-judgmental, patient-centered conversations about PrEP and HIV vulnerability that normalizes sexual health discussions that include PrEP as a preventive health tool, rather than basing recommendations solely on provider-assessed vulnerability or self-disclosed reasons for sexual health vulnerabilities. **Address PrEP Knowledge**: Educate cisgender Black women on PrEP to remove knowledge-based barriers to PrEP utilization, including information on PrEP availability at no-cost under almost all health insurance plans and Medicaid.
**Funders and** **Industry**	**Invest in Implementation Research:** Support PrEP studies that examine scalable models for integrating PrEP into primary and reproductive healthcare settings and ensure cisgender Black women’s participation in PrEP efficacy and adherence studies to develop data-driven strategies tailored to their needs. **Innovative Outreach Strategies:** Fund digital and community-based marketing campaigns that promote health communication models about PrEP knowledge among cisgender Black women and reframe PrEP narratives to align with Black women’s priorities for self-care, empowerment, and health autonomy; center Black women’s voices and experiences in PrEP marketing and outreach. **Support PrEP Distribution**: Allocate funding for innovative research on and implementation of community-based PrEP delivery models (e.g., mobile clinics, community spaces) to increase visibility of and access to PrEP in Black women’s communities.

## Data Availability

De-identified data can be made available upon request. Authors and Merck Pharmaceuticals maintain decision-making power as to how the data are shared.

## Appendix A

**Table A1:** Descriptive data describing responses to the eligibility screener compared to enrolled participants.

Descriptive Variables	Subcategories	All Respondents ^a^ (N = 216)		Study Participants (N = 20)	
Sex assigned at birth		N = 214	%	N = 20	%
	Female	209	98.12%	20	100.0%
	Male	4	1.88%		
	Missing	3			
Age		N = 205			
	<18 years	3	1.46%	0	0.0%
	18–29 years	146	71.22%	16	80.0%
	30–39 years	44	21.46%	3	15.0%
	40–49 years	7	3.41%	1	5.0%
	>49 years	5	2.44%	0	0.0%
Race		N = 202			
	Black/African American	202	100.0%	20	100.0%
Eligible county of residence		N = 198			
	Harris/Travis County	189	95.45%	20	100.0%
	Other County	9	4.55%	0	
County		N = 191			
	Harris County	107	56.02%	10	50.0%
	Travis County	84	43.98%	10	50.0%
Had condomless sex with a male partner within last 12 months		N = 189			
	Yes	147	77.78%	20	100.0%
	No	42	22.22%		
Taken PrEP within last 12 months		N = 147			
	Yes	32	21.77%		
	No	115	78.23%	20	100.0%
Prescribed PrEP within the last 12 months		N = 114			
	Yes	10	8.77%		
	No	104	91.23%	20	100.0%
Known HIV-positive status		N = 104			
	Yes	4	3.85%		
	No	100	96.15%	20	100.0%

*Note*. ^a^ All respondents represents all individuals who clicked the study link and started the eligibility screener.

**Table A2:** All themes and codes of facilitators to PrEP readiness with cisgender Black women identified through thematic analysis of focus group data from Houston and Austin, TX, USA.

#	Theme	Codes	Frequency of Codes
**Facilitators of PrEP uptake**			
**1**	**High perceived vulnerability**	***10 codes***	**204**
		Partner exposing participant to HIV vulnerability	58 **
		Self-awareness of one’s own HIV vulnerability behavior	39 *
		Concern about acquiring HIV	31 *
		Routine HIV resting	29 *
		Current engagement in HIV vulnerability behaviors	15
		Awareness that PrEP reduces vulnerability of HIV transmission in serodiscordant relationships	8
		Awareness of increased HIV vulnerability as a Black woman	7
		High vulnerability perceived with condomless sex	7
		Awareness of increased HIV vulnerability with multiple sex partners	6
		High perceived severity	4
**2**	**Current engagement in HIV prevention practices**	***4 codes***	**52**
		Current engagement in HIV prevention practices	32 *
		Ease of PrEP access	11
		Proactive about HIV vulnerability management (includes proactive about sexual health)	6
		Community engagement to promote PrEP	3
**3**	**Communal concern about Black women’s vulnerability to HIV**	***2 codes***	**22**
		PrEP offers community benefit	15
		Communal concern about Black women’s vulnerability to HIV	7
**4**	**Exposure to PrEP-related health communication**	***5 codes***	**99**
		Exposure to PrEP-related media	67 **
		Testimonials from Black women who take PrEP	13
		Normalize PrEP messaging through media	8
		Inclusivity in PrEP-related media	8
		Health communication to increase PrEP use and adherence	3
**5**	**Desire for access to humane treatment**	***3 codes***	**40**
		Educating and encouraging others to take PrEP	22
		Desire for transparency around PrEP	14
		Desire for access to humane treatment	4
**6**	**Prioritizing PrEP as self-care**	***6 codes***	**155**
		Prioritize PrEP as self-care	107 ***
		Sexual autonomy with prevention	21
		Sexual communication with partner	13
		Accountability for Black women to engage in preventative medicine	6
		Awareness that PrEP can reduce chances of acquiring HIV	6
		PrEP willingness driven by fear	2
**7**	**Preferences to ensure continuity of PrEP care**	***3 codes***	**182**
		Preferred modality	164 ***
		Ease of PrEP access	11
		Continuity of PrEP care	7
**8**	**Preferred modality**	***9 codes***	**176**
		Injection facilitator	82 **
		Daily pill facilitator	30 *
		Monthly pill as future PrEP option	14
		Clinician-administered injection	13
		PrEP patch as future option	13
		Preference for PrEP as a vaginal ring (includes vaginal ring as a future PrEP option, preference for a ring for PrEP, and PrEP as a vaginal ring)	10
		Desire for alternative modalities (includes desire for diversity of PrEP modalities)	6
		Gummy-style PrEP as future option	4
		Liquid PrEP as future option	4
**9**	**Preferences for engagement in PrEP**	***6 codes***	**84**
		Provider recommendation for health-related choices	38 *
		Desire for transparency around PrEP (duplicate of ‘need for transparency with pros and cons of PrEP use’)	14
		Desire for OBGYN to discuss PrEP options	12
		Trusted sources inform health-related decisions	12
		Desire for OBGYN to discuss PrEP options with women of color	5
		Community engagement to promote PrEP	3
**10**	**Positive experience with the healthcare system**	***2 codes***	**30**
		Positive experience with the healthcare system	16
		Desire for transparency around PrEP (duplicate of ‘need for transparency with pros and cons of PrEP use’)	14
**11**	**PrEP offers positive impacts on relationships**	***2 codes***	**17**
		Desire for transparency around PrEP (duplicate of ‘need for transparency with pros and cons of PrEP use’)	14
		Perception that PrEP would offer positive impact in relationships (includes code perception that PrEP use would have a positive impact on an intimate relationship)	3

*Note*. *** frequency of codes ≥ 100; ** frequency of codes ≥ 50; * frequency of codes ≥ 25.

**Table A3:** All themes and codes of barriers to PrEP readiness with cisgender Black women identified through thematic analysis of focus group data from Houston and Austin, TX, USA.

#	Themes	Codes	Frequency of Codes
**Barriers to PrEP uptake**			
**1**	**Low perceived HIV vulnerability**	***7 codes***	**117**
		Low perceived HIV vulnerability	50 **
		Certainty that PrEP is not an option	28 *
		Expectation of monogamy	23
		Low perceived severity	6
		No awareness of HIV burden to Black women	4
		Confidence in preventative measures outside of PrEP	3
		Choose not to view sexual health as a serious thing	3
**2**	**Negative experiences with healthcare system**	***4 codes***	**16**
		Structural barriers to shared decision-making with provider	6
		Lack of PrEP exposure by provider	4
		Negative experience with healthcare system	3
		Feeling disempowered by provider	3
**3**	**Medical mistrust**	***8 codes***	**64**
		Medical mistrust	32 *
		Unwillingness for PrEP due to historical trauma	8
		Current fertility concerns rooted in injustices in medical practice	6
		PrEP unwillingness linked to privacy concerns	6
		Condom as prevention option	5
		Concern about access to humane treatment	3
		Concern that discontinuing of PrEP will increase HIV vulnerability	3
		Use of COVID vaccine as example of communal injection aversion	1
**4**	**Lack of exposure to PrEP-related messaging for cis-hetero Black women**	***6 codes***	**78**
		Media messaging suggests PrEP is for LGBTQ+	39 *
		Representation needed by Black women as PrEP users	17
		Lack of exposure to PrEP media	9
		Lack of PrEP exposure by provider	4
		Perception that PrEP is for men based on TV media	4
		Unaware of PrEP modalities	5
**5**	**Concerns with continuity of PrEP care**	***10 codes***	**118**
		Concerns with PrEP contraindications or side effects	35 *
		Concern for pill side effects or interactions	22
		Burden to continuity of care	18
		Low perceived ability to adhere to daily pill	17
		Cost	9
		Concern with injection site	4
		Injection cost	4
		Distance to clinic	3
		Concern about long-term mental health implications	3
		Presumption that the pill’s way of preventing pregnancy applies to PrEP	3
**6**	**Concerns with modality**	***10 codes***	**210**
		Pill barriers	57 **
		Barrier to vaginal ring PrEP as future option	31 *
		Injection barriers	29 *
		Barrier to self-injection PrEP as future option	27 *
		Concern for pill side effects or interactions	22
		Low perceived ability to adhere to daily pill	17
		Concern for injection-related side effects	14
		Barrier to monthly pill as future option	5
		Injection cost	4
		Concern with injection site	4
**7**	**PrEP stigma**	***6 codes***	**97**
		PrEP stigma	49 *
		Perceptions of high vulnerability with PrEP use	27 *
		Family stigma around sex (formerly lack of family support due to stigma)	11
		PrEP unwillingness linked to privacy concerns (formerly PrEP unwillingness linked to lack of independence)	6
		PrEP use should not be linked to careless sexual activity	2
		Peers may assume a PrEP user has HIV	2
**8**	**Aversion to taking manufactured medication**	***6 codes***	**97**
		Concerns with PrEP contraindications or side effects	35 *
		Certainty that PrEP is not an option	28 *
		Concern for pill side effects or interactions	22
		Preference for holistic approach to health as a PrEP barrier	7
		Concern about long-term mental health implications	3
		Does not equate PrEP use with self-care	2
**9**	**Lack of PrEP knowledge as a barrier**	***3 codes***	**73**
		Lack of PrEP knowledge as a barrier	60 **
		Lack of exposure to PrEP media	9
		Lack of PrEP exposure by provider	4
**10**	**Concern that PrEP does not protect from other STDs**	***1 code***	**2**
		PrEP does not protect from other STDs (includes PrEP does not protect you from other infections)	2

*Note*. ** frequency of codes ≥ 50; * frequency of codes ≥ 25.
